# Evaluating the Readability, Accessibility, and Quality of Online Health Information on Opioid Use Disorder: A Comparative Analysis of Government and Non-Government Websites

**DOI:** 10.63116/SVXO9846

**Published:** 2026-02-12

**Authors:** Fatema Z. Ahmed, Alice Noblin, Kendall Courtelyou, Georgiana Tynes

**Affiliations:** 1 School of Global Health Management & Informatics, University of Central Florida

**Keywords:** Readability, accessibility, quality, health literacy, internet, information

## Abstract

**Background:**

The opioid crisis in the US remains a critical public health issue. Accessible, high-quality online health information is vital for educating the public about opioid use disorder (OUD) and available treatment options. This study aims to evaluate and compare the readability, accessibility, and quality of OUD-related information on government and non-government websites to identify areas for improvement.

**Methods:**

A total of 30 websites, 21 government-operated and 9 non-governmental, were selected through a systematic search. Readability was assessed using Gunning Fog, Simple Measure of Gobbledygook (SMOG), and Flesch Reading Ease Score (FRES) tests. Accessibility was evaluated using the WAVE tool, which checks for errors and adherence to Web Content Accessibility Guidelines (WCAG). Quality was measured using the DISCERN Instrument, a standardized tool for evaluating the quality of health information. Data were analyzed for statistical differences between government and non-government websites.

**Results:**

The average readability grade level across all websites was 14.63 and average FRES score was 31.63, indicating content requiring advanced reading skills, with no significant difference between government and non-government websites. Accessibility issues were more prevalent on non-government websites. Government websites scored significantly lower in terms of quality, with 80.95% of them rated as “poor” or “very poor” compared with 22.22% of non-government websites. The overall quality scores for both groups remained suboptimal, with an average DISCERN score of 39.52 out of 80.

**Conclusions:**

The findings highlight a critical need for improvements in the readability, accessibility, and quality of online health information on OUD. Government websites, in particular, require enhancements to ensure they meet the public's need for reliable, accessible, and comprehensible health resources.

## BACKGROUND

Opioid use disorder (OUD) has been declared a public health emergency in the United States due to its devastating impact on individuals, families, and communities.^1^ The crisis is marked by persistently high rates of misuse, dependence, and overdose mortality, with annual deaths nearly doubling between 2019 and 2022 to more than 81,000.^2^ Beyond mortality, OUD contributes to extensive health and social harms, including increased risk of infectious disease transmission,^3^ co-occurring mental health disorders,^4^ child welfare involvement,^5^ and criminal justice involvement.^6^ Addressing this crisis requires a comprehensive approach that integrates prevention, treatment, harm reduction, and recovery support, while also ensuring that the public has access to accurate, timely, and accessible information on opioid risks and evidence-based treatment options.

Public health interventions have increasingly focused on expanding access to evidence-based treatments by mitigating systemic barriers, including limited access to clinicians, insurance challenges, and inequities in the distribution of treatment resources. Despite these efforts, gaps remain; in 2021, approximately 2.5 million adults in the US were estimated to have opioid use disorder, yet only 36% received treatment, with an even smaller fraction (around 22%) receiving medications despite clinical guidelines that recommend the use of medications for OUD.^7^

Online health information is a crucial asset in combating the opioid epidemic by facilitating better public education, expanding access to treatment resources, and fostering community engagement, all of which are necessary components in reducing opioid-related harm. Effective public information and communication strategies are critical to reducing stigma, shaping public attitudes, and increasing treatment engagement.^8–10^

Despite the growing availability of online resources for OUD, there is a scarcity of research that systematically assesses the readability, accessibility, and quality of webbased information. Studies have consistently shown that online health materials across topics are written above recommended reading levels, limiting their utility for the general public.^11–13^ In the context of OUD, the evidence base is particularly limited a few studies highlight readability concerns in naloxone (an opioid overdose reversal medication) and OUD-related websites,^14,15^ while others point to the spread of misinformation related to medications for OUD.^16^ Accessibility has also been underexamined in OUD research, though broader evaluations of health websites demonstrate persistent gaps, including barriers for people with disabilities.^17,18^

Notably, there is a particular lack of research directly comparing government versus non-government websites, despite both being key sources of information for the public. Government-based websites hold a unique responsibility in this landscape due to their role in disseminating accurate, high-quality, and inclusive information. Given their public trust, legal obligations, and ethical mandates, these sites should prioritize readability, accessibility, and reliability, as they are crucial for ensuring equitable access to health information across diverse populations. Historical research suggested that government websites were often more accessible than privately operated ones due to compliance requirements, yet still fell short of optimal standards.^19,20^

More recent work underscores these concerns. Analyses of state and territorial public health websites and patient education resources show poor accessibility and readability,^21,22^ provincial government messaging has been found to exceed recommended readability thresholds,^23^ and even international and federal agency websites have been criticized for overly complex language.^24^ These findings indicate that while government websites may in some respects be more accessible than their counterparts, neither consistently reaches acceptable levels of readability or accessibility. Therefore, the objective of this study was to systematically evaluate the readability, accessibility, and quality of online health information on OUD, with a particular emphasis on comparing government-operated websites with non-government websites. This evaluation aims to provide insights into the current state of online health information and identify areas for improvement to better serve the public in addressing the opioid epidemic.

### Health Literacy

Health literacy remains a significant public health concern in the United States, with many Americans struggling to understand and use health information effectively. According to the 2023 U.S. Program for International Assessment of Adult Competencies (PIAAC) report, 28% of adults scored at or below Level 1, indicating they could understand only simple sentences.^25^ In contrast, only 44% reached Level 3 or higher, the threshold considered the minimum for full proficiency. Alarmingly, the proportion of adults scoring below or at a Level 1 has risen from 19% in 2017 to 28% in 2023, highlighting a notable decline in adult literacy over time.^25^ Populations disproportionately affected include racial/ethnic minorities and individuals with lower educational attainment.^26^ Limited health literacy is associated with poorer health outcomes, increased hospitalizations, higher mortality, and reduced use of preventive services.^27–29^

Inadequate health literacy affects nearly 88% of U.S. adults, limiting their ability to effectively navigate the health system and optimize their health outcomes.^30^ Health literacy disparities also influence preventive health behaviors and self-rated health status among older adults.^26^ Addressing health literacy by simplifying communication and tailoring information to the cultural, linguistic, or community context can reduce disparities and improve health outcomes.^30,31^

### Online-Based Health Information/Misinformation

Online-based health information is crucial due to its accessibility, convenience, cost-effectiveness, and empowering potential. In 2022, 58.5% of adults sought health or medical information online.^32^ Online platforms provide immediate access to diverse formats, including articles, videos, infographics, and interactive tools, catering to various learning styles and preferences. Additionally, online forums and support groups offer emotional support and practical advice for individuals with health conditions. These platforms also facilitate access to the latest research, clinical trials, and medical advancements.

However, the prevalence of online health misinformation poses a significant challenge. Research highlights that such misinformation often includes negative sentiments, anecdotal evidence, and anti-science narratives.^33^ Social media accelerates the spread of these inaccuracies, potentially harming public health.^34^ Persuasive strategies in misinformation include fabricating narratives, using personal anecdotes, and distrusting authorities.^35^ Factors such as health-related anxiety, preexisting misinformation beliefs, and repeated exposure contribute to the acceptance of false information, with demographic variables like gender, age, and socioeconomic status also playing a role.^36^

Despite the prevalence of misinformation, several sources are generally considered reliable for online health information. These include government agencies, medical institutions, and recognized health organizations, which adhere to rigorous accuracy standards. However, research indicates that government websites are not always immune to inaccuracies or unclear information.^37^ Government- funded sites, while typically more readable than commercial ones,^38^ sometimes disseminate unvetted third-party content that may be misleading.^39^ During public health crises, government agencies and news media are generally more effective at correcting misinformation compared to social peers.^40^ Thus, while government websites offer certain advantages, they are not exempt from the risk of health-related misinformation.

### Readability of Online-Based Health Information

Online health information often exceeds recommended readability levels, potentially compromising public understanding. Studies have found that health-related websites in the United States and Canada have mean readability grades ranging from 10 to 15, which is significantly higher than the recommended 6th-grade level.^11,41^ This issue persists across various health topics and organizational types.^13,42,43^ Government-funded websites tend to be slightly more readable than those funded by the private sector, although both still fall short of ideal levels.^24,38^ This is problematic because high readability levels can contribute to misinformation and adverse health outcomes.

### Accessibility of Information Online

Accessibility of online health information is crucial for promoting health equity and improving outcomes, yet significant barriers persist. A study evaluating 139 health-related websites reported that 91% contained accessibility issues, with government websites ranking third in overall error density.^17^ In a separate review of 157 US children's hospital websites, none were found to be fully compliant with established web accessibility guidelines.^44^ Similarly, an assessment of 106 leading hospital websites revealed that only 4.9% fully met the Web Content Accessibility Guidelines (WCAG), a set of internationally-recognized standards developed by the World Wide Web Consortium (W3C) to make web content accessible to people with disabilities.^18^ Additional evaluations of major health information platforms have documented widespread navigation challenges for users with disabilities.^45^ These findings are especially concerning in light of recent federal guidance. The US Department of Justice's 2022 *Americans with Disabilities* Act (*ADA) Web Accessibility Guidance*^46^ and the US Department of Health and Human Services' Office of Civil Rights Guidance on Nondiscrimination in Telehealth^47^ both reaffirm that public-facing websites, including those delivering health information, are subject to the ADA and must meet accessibility standards such as WCAG. Together, these studies and policies underscore the urgent need to improve web accessibility, ensuring equitable access to online health information for all users, including individuals with disabilities and older adults.

### Quality of Online-Based Health Information

The quality of online health information is crucial, as it can significantly influence users' health decisions and behaviors.^48,49^ Research evaluating health websites using various criteria has shown mixed results: while some studies report high-quality information,^48,50^ others indicate that overall quality remains suboptimal and unreliable for laypersons.^50^ Quality varies by health topic, website affiliation, and medical specialty,^48,50^ with government and academic sites generally providing higher-quality content.^51^ The emphasis on user-centered design and socio-technical aspects in quality assessments highlights their growing importance.^52^ Despite these efforts, online health information often remains inconsistent and can be misleading, making it challenging for consumers to discern its credibility.^53^

### Study Objective

This study aims to systematically assess the readability, accessibility, and quality of online health information concerning OUD, with a specific focus on comparing government-operated websites wit non-governmental websites. Research examining online information on OUD, while very limited, reveals concerns about readability, usability, quality, and misinformation. Several studies have found that OUD-related websites and educational materials often exceed recommended readability levels, with content written at a level above college, making it difficult for many users to understand.^54,55^ Usability analyses have shown that OUD websites frequently lack user-friendly navigation and accessibility features, posing barriers for people seeking help.^15^ In addition, studies analyzing video content and social media platforms (eg, YouTube, Reddit) have identified widespread dissemination of low-quality information and persistent myths about OUD and treatment.^55–57^

However, to date, no research has simultaneously examined three dimensions (readability, accessibility, and quality) in the context of online health information regarding OUD specifically. Furthermore, no study has compared these dimensions between government and non-government websites. This investigation seeks to elucidate the current state of online health information regarding OUD and to identify areas for potential improvement.

## METHODS

### Search Strategy

In January 2024, we conducted internet searches using keywords related to OUD: “opioid addiction,” “opioid dependence,” and “opioid abuse.” We analyzed the initial 30 English-language websites resulting from each query, mirroring the approach of an average user with limited medical or internet literacy.^58^ Exclusions comprised sponsored links, news articles, inaccessible sites requiring payment or membership, sites lacking pertinent information on opioid addiction/abuse/dependence, discussion groups or forums, non-website sources like books or videos, and non-English content.

### Sample

We assessed ninety websites in total. Significant overlap existed among the websites yielded by each search term, resulting in 63 distinct websites. Subsequent exclusions, totaling 26 websites, were warranted for various reasons: 17 were deemed non-website entities (ie, links to books, videos, news articles, or discussion groups), 1 was inaccessible, 2 were sponsored links, and 6 were found to lack pertinent information. During the various analytical tests for readability, accessibility, and quality, 7 websites resulted in errors and were therefore excluded from the final sample; consequently, our final sample comprised 30 websites. Twenty-one of the websites were government websites (ie, had a suffix of .gov), and 9 were other classifications of websites (ie, “.com,” “.org,” “.int”). Most of the websites resulted from the search term “opioid addiction.” See Table 1 for complete sample characteristics.

**Table 1.** Sample Characteristics

**Website Suffix**	**Percent (n)**
.ca	3.33 (1)
.com	3.33 (1)
.gov	70.00 (21)
.int	3.33 (1)
.org	20.00 (6)

### Evaluation of the Websites

Websites were evaluated for their readability, accessibility, and quality. Results were compared between government websites and non-government websites.

#### Readability

Text readability is “the determination by systematic formulae of the reading comprehension level a person must have to understand written materials.”^59^ This investigation evaluated readability using Readability Studio Professional Edition, Version 2024 (Oleander Software Ltd; Vandalia, Ohio). Three distinct instruments were employed to assess readability. These comprised two reading grade level tests, (Gunning Fog [FOG] and Simple Measure of Gobbledygook [SMOG]), and one index score test (Flesch Reading Ease Score [FRES]). The FOG^60^ and SMOG^61^ tests estimate the years of education needed to comprehend a passage of text based on factors such as the number of characters, syllables, words, or sentences in a text sample and have been widely used to assess the readability of health information.^43,62,63^ For instance, a text with a FOG/SMOG score of 8 would be understandable to an 8th-grade student. Scores higher than 12 (ie, 12^th^ grade) suggest the text is quite challenging to read and comprehend; therefore, it falls into the “college level” or “beyond college level” categories, requiring a high level of education to achieve a complete understanding. The FRES test computes a text's readability as an index score based on factors such as sentence length and syllable count.^64^ This score ranges from 0 to 100, with higher values indicating greater ease of comprehension. A score of 90-100 is considered “very easy” or at a 5^th^ grade level, 60-70 is considered “standard” or 8^th^-9^th^ grade level, 30-50 is considered “difficult” or 10^th^-12^th^ grade level, and 0-30 is considered “very difficult” or college level. This scale is widely used to evaluate health information.^50,65^

#### Accessibility

Web accessibility ensures that all users, including those with disabilities, can perceive, understand, navigate, interact with, and contribute to websites without encountering barriers to accessing content like text, images, audio, video, or multimedia. Accessibility guidelines, such as the Web Content Accessibility Guidelines (WCAG) established by the World Wide Web Consortium (W3C), provide technical requirements for making digital content accessible and usable.^66^ To assess web accessibility, we employed the WAVE automatic accessibility testing tool.^67^ WAVE is designed to help developers, content creators, and researchers assess the accessibility of web pages, particularly in alignment with WCAG, by identifying accessibility errors, alerts, and features that may affect how well users, especially those with disabilities, can navigate and understand the page. Its evaluation generates a report outlining errors (eg, missing alternative text, empty buttons or links), alerts (eg, small font sizes, long link texts), features (eg, properly structured headings), contrast issues (eg, contrast ratio standard), structural element problems (eg, semantic structures like headings and lists), and usage of HTML5 and Accessible Rich Internet Applications (ARIA), a set of attributes that improve the accessibility of web content, within a webpage.

#### Quality

The DISCERN Instrument helps consumers and information providers assess the quality of health information. The DISCERN Handbook ensures all users understand and apply the instrument effectively.^68^ It consists of 16 questions covering clarity, balance, and content, each representing a separate quality criterion. Questions are rated on a 5-point scale (1 = definite ‘no’; 2-4 = ‘partially meets criteria’; 5 = definite ‘yes’). The first eight questions assess the reliability of publication, while the last seven address details of treatment information. The final question offers an overall quality rating. A website's quality, as assessed by the DISCERN instrument, is classified according to its total score: 63-80 indicates “excellent” quality, 51-62 “good,” 39-50 “fair,” 27-38 “poor,” and 15-26 “very poor.”

### Data Analysis

#### Readability

FOG, SMOG, and FRES scores were calculated for each website, as using multiple tools enhances confidence in evaluations by covering different dimensions and reducing bias. To simplify interpretation, a single reading grade (RG) was derived for each website by averaging the FOG and SMOG scores (similar to a previous study^43^). T-tests were used to determine significant differences in RG between government and non-government websites. Statistical analysis was performed using the Stata software (v.17).^69^

#### Accessibility

The WAVE tool evaluates web content for conformance with WCAG 2.1 Levels A and AA, which represent the most widely adopted accessibility standard and are often required by law under statutes such as the Americans with Disabilities Act (ADA) and Section 508 of the Rehabilitation Act. Univariate analyses (ie, measures of central tendency) were conducted on the data using Stata Software (v.17).^69^

#### Quality

Final DISCERN scores were calculated by summing the ratings across all 16 items, with higher scores indicating greater quality. Two researchers independently assessed each website, and the average of their total scores was used for analysis to ensure consistency and reduce bias. Wilcoxon rank sum tests were conducted to analyze the differences in quality scores between government and nongovernment websites using Stata software (v.17).^69^

## RESULTS

### Readability

A single reading grade (RG) was created for each website by averaging the FOG and SMOG scores. To justify this approach, correlations among the readability measures were examined. FOG and SMOG were strongly correlated (r = 0.8437, p < 0.0001). The RG also showed strong correlations with both FOG (r = 0.9627, p < 0.0001) and SMOG (r = 0.9575, p < 0.0001), and a strong negative correlation with FRES (r = −0.6904, p < 0.0001). The analysis of 30 websites yielded an average RG of 14.63, indicating that the content was written above the college level. The average FRES was 31.63. The FRES range from 1 to 100, with higher scores indicating greater readability. We found that government websites had an average RG of 15.00 and a FRES of 30.38, while non-government websites showed an average RG of 13.74 and a FRES of 34.56. These findings suggest that most websites assessed contain content that is likely too complex for the average reader to comprehend. There was no significant effect on RG by website type (*t*[28] =−1.77, *p* = 0.087), despite government websites (*M* = 15.0, *SD*=1.71) attaining higher reading grade levels than non-government websites (M=13.74, SD=1.98). Table 2 shows complete readability results.

**Table 2.** Average Reading Grade and Reading Ease

	**Overall Sample**	
	*Mean*	*SD*
RG*	14.63	1.85
FOG	14.27	1.99
SMOG	14.98	1.87
FRES	31.63	11.97

	By Website Type	
	*Government* *Mean (SD)*	*Non-Government* *Mean (SD)*
RG*	15.00 (1.71)	13.74 (1.98)
FRES	30.38 (9.07)	34.56 (17.31)

Note: *Reading grade is the average of the FOG and SMOG scores.

FOG, Gunning Fog Index; FRES, Flesch Reading Ease Score; SMOG, Simple Measure of Gobbledygook; RG, Reading Grade.

### Accessibility

The WAVE tool evaluates website accessibility across six categories: (1) Errors, (2) Alerts, (3) Structural elements, (4) Contrast, (5) Features, and (6) ARIA. Among the 30 websites analyzed, 12 (40%) passed without any errors, all of which were government websites. Across all websites, 149 errors were detected, with an average of 4.967 errors per site and a maximum of 63 errors. Ten websites (33.3%) exceeded the average of 4 errors. The most common issues were related to structural elements (26%) and ARIA compliance (54%). Non-government websites exhibited a higher mean number of total errors (M = 308.5, SD = 218.7) compared to government websites (M = 203, SD = 230.7). Table 3 shows complete accessibility results.

**Table 3.** WAVE tool Accessibility Results

	**Government Websites (n-21)**							**Non-Government Websites (n=9)**						
	Error	Alerts	Structural	Contrast	Features	ARIA	*Total*	Error	Alerts	Structural	Contrast	Features	ARIA	*Total*
1.	0	21	17	30	8	57	133	5	8	61	0	18	152	244
2.	0	5	48	2	4	68	127	3	7	75	0	23	279	387
3.	5	66	45	0	5	317	438	1	25	138	0	15	442	621
4.	14	7	116	0	9	33	179	1	13	45	4	11	13	87
5.	0	33	47	7	28	46	161	14	81	70	4	13	13	195
6.	0	6	22	0	26	14	68	63	50	99	0	16	291	519
7.	0	15	39	2	24	40	120	1	8	60	2	51	49	171
8.	0	8	124	6	13	101	252	1	2	1	0	1	0	5
9.	0	6	37	0	11	69	123	9	66	99	19	32	323	548
10.	5	18	38	2	23	43	129							
11.	0	10	48	1	14	27	100							
12.	2	17	55	3	34	58	169							
13.	1	38	156	0	9	944	1148							
14.	5	3	34	0	7	18	67							
15.	7	13	47	16	15	26	124							
16.	0	6	32	1	11	69	119							
17.	4	17	50	0	10	71	152							
18.	0	6	35	1	10	36	88							
19.	8	31	28	5	29	48	149							
20.	0	37	77	0	47	75	236							
21.	0	26	66	0	35	54	181							
*Mean*	2.429	18.524	55.286	3.619	17.714	105.429	203	10.889	28.889	72	3.222	20	173.556	308.556
*SD*	3.749	15.52	35.453	7.124	11.735	201.857	230.709	20.053	29.328	38.506	6.16	14.361	164.683	218.693

	**Overall Sample (n=30)**						
Error	Alerts	Structural	Contrast	Features	ARIA	*Total*							
*Total*	149	649	1809	105	552	3776	7040
*Mean*	4.967	21.633	60.3	3.5	18.4	125.867	234.667
*SD*	11.669	20.657	36.559	6.745	12.37	191.287	228.74

### Quality

The two researchers' total scores were averaged to enhance consistency and reduce individual scoring bias. Their scores demonstrated a strong correlation (r = 0.723, p < 0.0001), supporting the decision to use the averaged value in analyses. Out of the thirty websites evaluated, 11 (36.67%) achieved a total DISCERN score of 39 or higher, classifying them as “fair” or above. The mean DISCERN score was 39.52 out of 80, which corresponds to a “fair” ranking. However, the majority of websites (56.67%) were rated as “poor.” Specifically, 80.95% of government websites were ranked as “poor” or “very poor,” compared to 22.22% of non-government websites. A Wilcoxon rank sum test revealed that the median quality scores were statistically significantly lower for government websites than for non-government websites (Z = 2.67, p < .01). Table 4 shows complete quality results.

**Table 4.** Quality Rankings

	Overall Sample (n=30)	Non-Government Websites (n=9)	Government Websites (n=21)
Quality Ranking	Percent (n)	Percent (n)	Percent (n)
Excellent	3.33 (1)	11.11 (1)	0 (0)
Good	13.33 (4)	33.33 (3)	4.76 (1)
Fair	20.00 (6)	33.33 (3)	14.29 (3)
Poor	56.67 (17)	22.22 (2)	71.43 (15)
Very poor	6.67 (2)	0 (0)	9.52 (2)

Note: Websites were classified based on their total scores as ‘excellent' (63–80), ‘good' (51–62), ‘fair' (39-50), ‘poor' (27–38), or ‘very poor' (15–26).

## DISCUSSION

Readability, accessibility, and quality of online health information are crucial for informed decision-making, equitable access, and improved health outcomes. Studies have consistently found that online health information is written at a level too complex for the general public, requiring high school or higher reading ability.^11,70^ This poor readability can lead to misinformation and potentially harm patients' health.^11^ Accessibility issues also exist, with search engines often providing inefficient results and many websites lacking comprehensive coverage of key clinical information.^70^ Factors influencing readability include the health condition, website domain, and paragraph position within texts.^43^ Improving the readability and accessibility of online health information is essential to enhance health literacy and ensure equitable access to healthcare services.

Our findings indicate that the majority of websites analyzed in this study had a reading grade level exceeding that of a college education, reflecting a high degree of complexity in the website's content. This observation is consistent with previous research, which has similarly identified that the readability of health-related websites often far surpasses the recommended 6th-grade level.^11,41^ While prior studies have suggested that government websites tend to be marginally more readable than their non-government counterparts,^24,38^ our analysis revealed that government websites were actually less readable, though the difference was not statistically significant. These results suggest that many websites are written at a level higher than the average American reading proficiency. This can adversely affect users' understanding of the information and, in turn, impact their decision-making and expectations. Moreover, research shows that individuals with OUD have lower levels of education than the general population.^71^ Therefore, these results are even more detrimental to the population that is most likely to search for OUD-related information online.

Research has consistently highlighted readability issues in online health information.^11,12,14,72,73^ The ongoing prevalence of these readability problems is a matter of significant concern. It is particularly alarming that government websites—designed to be accessible to the public—continue to present information in a manner that may obstruct understanding, especially regarding critical topics such as opioid use disorder. This ongoing issue highlights a significant barrier to effective communication. It underscores the need for improvement in the readability and accessibility of online health information to ensure that it meets the needs of the entire population.

Our analysis revealed an average of 4.967 accessibility errors per website, with non-government websites exhibiting a higher error rate than government sites. While our findings align with existing literature,^17,74–76^ which suggests that government websites are generally more accessible, it is noteworthy that many errors on these sites were related to ARIA compliance issues. ARIA attributes, defined by the W3C, are crucial for enhancing web content accessibility, particularly for dynamic elements and advanced user interface controls. Significant ARIA errors indicate impaired accessibility and unequal information access. This is particularly significant considering the 2022 US DOJ and DHHS web accessibility guidelines, which reaffirmed that public-facing websites, including those operated by government agencies, must comply with the ADA and meet recognized accessibility standards such as the WCAG, which include proper ARIA implementation.^46,47^ The persistence of ARIA-related errors, even on government websites, suggests gaps in full compliance and highlights the need for ongoing monitoring and remediation to uphold legal and ethical standards for digital accessibility. Persistent accessibility issues may damage the reputation of government agencies, eroding public trust in their commitment to inclusivity.

Our analysis revealed that 56.67% of websites were rated as “poor” quality, with a notable 80.95% of government websites falling into the “poor” or “very poor” categories, substantially higher than the 22.22% of non-government websites rated similarly. Low-quality online health information poses significant risks, including the potential for users to make incorrect health decisions, which may result in harmful outcomes or ineffective treatments. Such information can promote unsafe practices or unproven remedies, increasing the risk of adverse health effects. Persistent exposure to misleading content can erode trust in online health resources and healthcare professionals, contributing to the spread of misinformation, particularly on social media. This can impair users' health literacy and lead to disparities in care for those who rely on online information without proper professional guidance. Ensuring that online health information is accurate, evidence-based, and clearly presented is crucial for informed decision-making, effective self-care, and maintaining public trust in health resources.

It is crucial to recognize that not all websites are designed for a general audience. For instance, academic texts cater to a different readership than government websites or charities that aim to disseminate health information. The diversity of website types appearing on the first page of search results is expected, as various entities compete to promote their content. Given that online information increasingly serves as the primary source for many individuals, it is essential that the information featured prominently in search results is both accessible and accurate, even though achieving such accessibility and accuracy is challenging due to the internet's rapidly evolving nature.

### Limitations and Future Research

Our study focused on analyzing the first 30 search results on Google for three specific search terms. Future research should consider larger sample sizes and include other popular search engines to enhance the robustness of the findings. Additionally, the results reflect the content available at the time of data collection, and given the dynamic nature of the internet, these findings may not be applicable at other times.

The DISCERN quality assessment tool, while useful for evaluating the quality of online health information, may be less effective for assessing other types of website content. For instance, many websites in our sample did not emphasize treatment options for OUD, though the DISCERN tool includes several questions on treatment, potentially skewing the overall quality scores. Therefore, evaluating the accuracy of online health information content is also recommended.

Our study did not examine user-generated content or social media platforms, which are increasingly significant sources of health information. Future research should address these sources to provide a more comprehensive view. Lastly, our analysis was limited to English-language websites, yet the US population is diverse and includes many non-English speakers. Exploring non-English websites could reveal similar issues in other languages.

## CONCLUSIONS

Our study reveals that websites on OUD are generally difficult to read, access, and of low quality, with government websites performing poorer than non-government sites in these areas. Given the pivotal role of online information in public health crises, there is an urgent need to improve and update websites addressing opioid use disorder. Online platforms are crucial for disseminating information on the risks of opioid misuse, signs of addiction, treatment options, and contact details for support services. They also facilitate emotional support and connection through online communities and forums. It is especially important for government websites to meet high standards of readability, accessibility, and quality, given their perceived trustworthiness due to regulatory oversight, expert input, and public accountability.

## DISCLOSURES

The authors have nothing to disclose.

## FUNDING SOURCES

The authors received no funding for this research.
